# Hypercalcemia in Dual Pathology: Interplay Between Renal Cell Carcinoma and Parathyroid Adenoma

**DOI:** 10.7759/cureus.67095

**Published:** 2024-08-17

**Authors:** Maya Mazuwin Yahya, Nur Karyatee Kassim, Tuan Salwani Tuan Ismail, Jun Jun Thien, Irfan Mohamad

**Affiliations:** 1 Department of Surgery, School of Medical Sciences, Universiti Sains Malaysia, Kubang Kerian, MYS; 2 Basic and Medical Sciences Unit, School of Dental Sciences, Universiti Sains Malaysia, Kubang Kerian, MYS; 3 Department of Chemical Pathology, School of Medical Sciences, Universiti Sains Malaysia, Kubang Kerian, MYS; 4 Department of Pathology, Sarawak General Hospital, Kuching, MYS; 5 Department of Otorhinolaryngology-Head and Neck Surgery, School of Medical Sciences, Universiti Sains Malaysia, Kubang Kerian, MYS

**Keywords:** renal cell carcinoma, parathyroid adenoma, intraoperative parathyroid hormone, hypercalcemia, primary hyperparathyroidism

## Abstract

The concomitant occurrence of renal cell carcinoma (RCC) and primary hyperparathyroidism is rare as these conditions are often identified by the presence of hypercalcemia, which might be missed in asymptomatic individuals. We present the case of a 58-year-old asymptomatic male detected to have a left abdominal mass during his routine medical follow-up. He was subsequently diagnosed with RCC. Further history revealed that his calcium levels had been persistently elevated for the past eight years but had never been investigated. Based on elevated parathyroid hormone levels and radiological findings, a diagnosis of primary hyperparathyroidism has also been made. A right inferior parathyroidectomy was performed, and the histopathological examination results showed a right parathyroid adenoma. Intraoperative intact PTH (iPTH) measurements confirmed the complete removal of the abnormal parathyroid gland. The postoperative calcium levels have returned to normal. To the best of our knowledge, this was the first reported case of concurrent primary hyperparathyroidism and RCC in our population. This case illustrates the importance of considering a broad differential when evaluating patients with hypercalcemia.

## Introduction

The occurrence of concomitant diseases in an individual is rare. Therefore, in the case of malignancy-associated hypercalcemia, both primary and secondary causes must be carefully investigated in clinical practice to avoid missing out on the presence of concomitant primary and secondary causes of hypercalcemia. Hypercalcemia is associated with a few primary cancers and various metastatic cancers [1]. The differential diagnoses to be considered when investigating hypercalcemia include both primary causes, such as parathyroid adenoma or tertiary hyperparathyroidism, and secondary causes, such as paraneoplastic syndrome or bone metastases [2]. Despite the rare occurrence of concomitant primary and secondary causes in a patient, delineating both causes in each patient is crucial [3]. A proper assessment of hypercalcemia is crucial for identifying the causes, deciding the appropriate management, and preventing its complications.

## Case presentation

A 58-year-old patient with underlying type II diabetes mellitus (T2DM), hypertension, and dyslipidemia was found to have a left abdominal mass during his routine medical follow-up. He was otherwise asymptomatic with no abdominal pain. An abdominal ultrasound (USG) of the abdomen identified a left renal mass and a right renal stone, while further renal imaging by high-dose four-phase contrast-enhanced computed tomography (CECT) revealed a well-encapsulated left renal mass suggestive of renal cell carcinoma (RCC). A laparoscopic left nephrectomy was performed, and the result of histopathological examination (HPE) showed left RCC Fuhrman grade 1. Additionally, a positron emission tomography (PET) scan conducted 10 days after the operation showed no evidence of metastatic lesion.

A preoperative investigation revealed hypercalcemia, as shown in Table 1, and further history indicated that his calcium levels had been persistently elevated for the past eight years despite primary care follow-up; however, they were not investigated because of his asymptomatic status. His calcium levels ranged from 2.4 mmol/L to 4.0 mmol/L, and because of persistent hypercalcemia, serum intact parathyroid hormone (iPTH) was assessed preoperatively, demonstrating an elevated level of 58.1 pmol/L, with a correspondingly low serum phosphate concentration of 0.63 mmol/L. Parathyroid hormone-related peptide (PTHrP) was not measured.

**Table 1:** Preoperative biochemical laboratory findings. iPTH: intact parathyroid hormone, TSH: thyroid-stimulating hormone, T4: thyroxine

Analytes	Preoperative	Reference range
Serum iPTH (pmol/L)	58.1	1.6–6.9
Serum calcium (mmol/L)	2.79	2.2–2.6
Serum albumin (g/L)	36	38–44
Corrected serum calcium (mmol/L)	2.87	2.2–2.6
Serum phosphate (mmol/L)	0.63	0.84–1.45
Serum TSH (mIU/L)	1.97	0.27–0.42
Free T4 (pmol/L)	14.57	12–22
Serum creatinine (mmol/L)	447	2.02–2.60
Serum urea (mmol/L)	17.2	1.7–8.3

**Figure 1:**
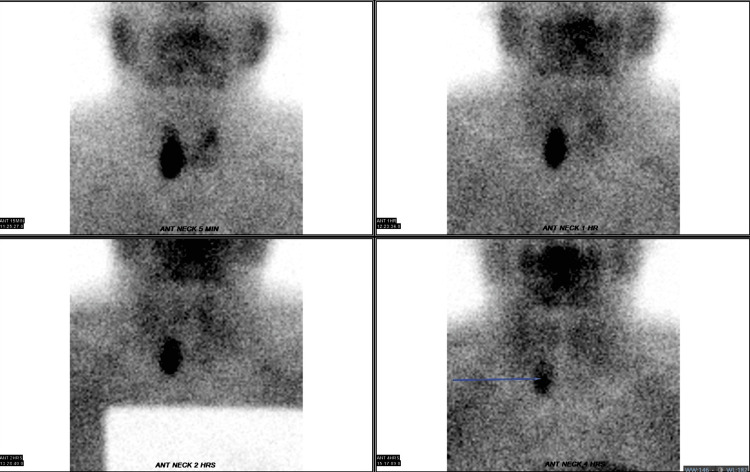
Parathyroid scintigraphy showing a lesion suggestive of functioning parathyroid adenoma at the inferior pole of the right thyroid lobe (blue arrow).

**Figure 2:**
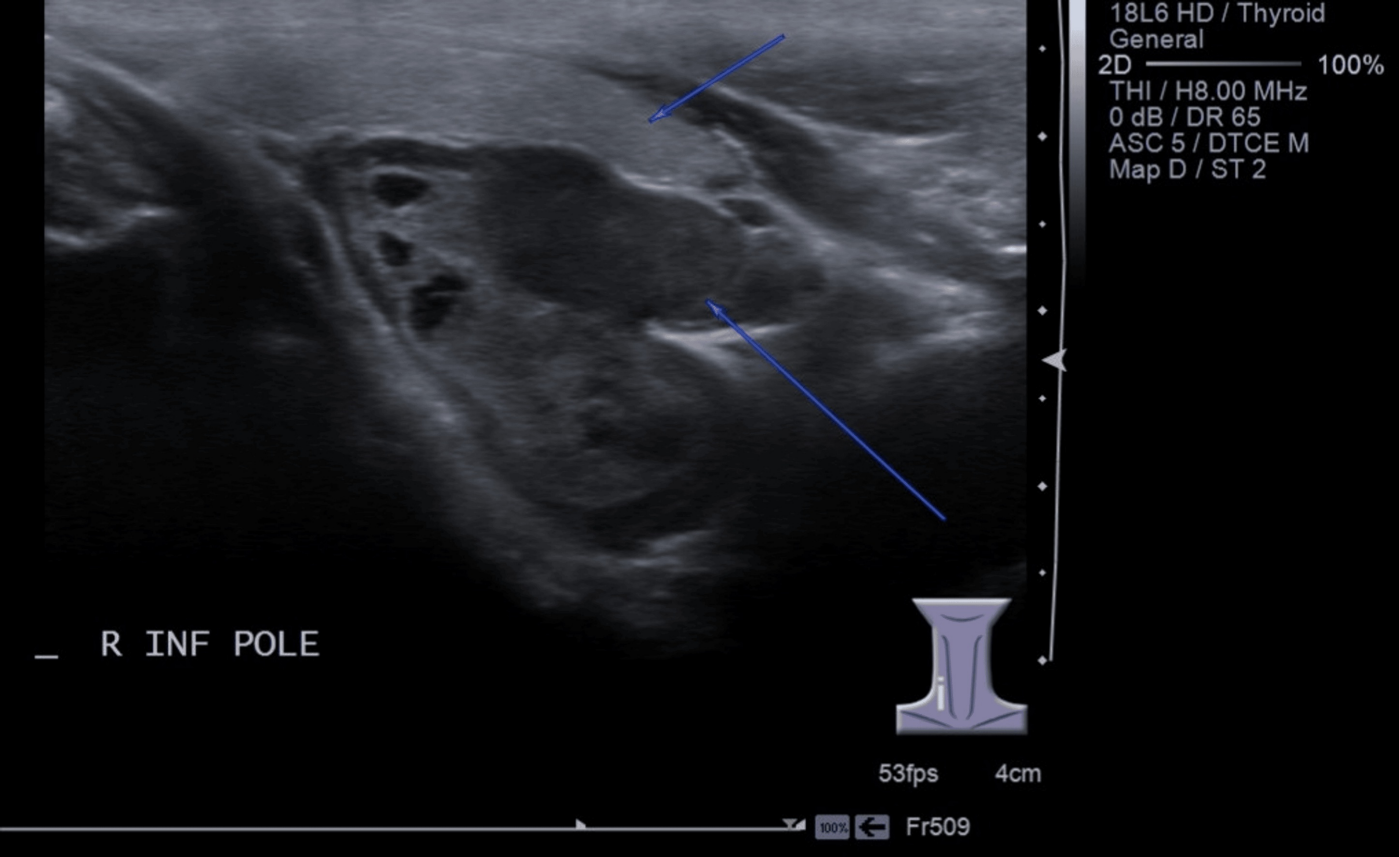
Ultrasound scan (longitudinal) demonstrating well-defined mixed echogenicity solid cystic lesion located posterior to the inferior pole of the right thyroid gland. The short blue arrow shows a homogenous right thyroid lobe, and the long blue arrow shows a heterogeneous lobulated parathyroid nodule.

Further imaging with technetium-99m (Tc-99m) sestamibi scintigraphy identified a functioning parathyroid adenoma at the inferior pole of the right thyroid lobe (Figure 1). Neck USG findings were consistent with a Tc-99m sestamibi scan, revealing a right parathyroid adenoma measuring 1.9 cm x 1.9 cm x 2.9 cm (anteroposterior x width x craniocaudal (AP x W x CC)), a dominant thyroid nodule on the left and a bilateral calcified plaque on the common carotid arteries (Figure 2).

He underwent a left hemithyroidectomy and a right inferior parathyroidectomy for a left thyroid cyst, as well as a right parathyroid. The left thyroid cyst, measuring 4 cm × 4 cm, and the right inferior parathyroid gland, measuring 2 cm × 2 cm, were removed. Intraoperative iPTH (IOPTH) measurements confirmed the complete removal of the abnormal parathyroid gland (Table 2). Postoperatively, the corrected calcium levels decreased within the normal range, from 2.12 mmol/L to 2.3 mmol/L. The patient was discharged four days after the operation and scheduled for follow-up at the endocrine clinic. HPE confirmed the presence of a right parathyroid adenoma and left colloid nodules.

**Table 2:** Reduction of serum parathyroid hormone level of more than 50% at 10 and 20 minutes post-parathyroid gland removal.

Analyte	Zero-minute pre-skin excision	10 minutes post-parathyroid gland removal	20 minutes post-parathyroid gland removal
Serum iPTH (pmol/L)	107.3	10.5	6.2

## Discussion

We present a case of asymptomatic hypercalcemia in a patient with RCC and concomitant primary hyperparathyroidism. Hypercalcemia is a metabolic disorder that can result from various conditions, with primary hyperparathyroidism as the predominant cause. Typical symptoms of hypercalcemia include generalized bone disorders, renal manifestations, constipation, and neuropsychiatric symptoms. Most patients are usually asymptomatic and are identified by routine biochemical tests based on serum PTH and calcium levels [4]. Hypercalcemia and its complications significantly impact patients’ quality of life, including fatigue, muscle weakness, cognitive impairments, and gastrointestinal disturbances such as nausea and constipation, all of which can severely limit daily activities and overall well-being. Therefore, it is essential to identify and treat adequately the etiology of hypercalcemia in cancer patients, including those with advanced disease. Isolated primary hyperparathyroidism is the cause of hypercalcemia in 5% to 10% of cancer patients. However, the association of primary hyperparathyroidism with humoral hypercalcemia of malignancy is rare [3].

Hypercalcemia develops in approximately 17% of patients with RCC during their disease course, the most likely cause of which in RCC is because of the paraneoplastic phenomenon [2]. A protein called PTHrP is elevated in up to 47% of patients, with malignancy and hypercalcemia [3]. In this case, serum PTH levels are usually suppressed, and the serum phosphate level usually ranges from low to normal. The PTHrP level measurement is not routinely performed, as in our case, because of its prohibitive cost and limited assay availability. Other plausible causes of hypercalcemia in RCC include lytic bone lesions, which were ruled out in this patient based on a PET scan. In this case, the combination of elevated serum parathyroid levels, hypercalcemia, and hypophosphatemia prompted an initial suspicion of primary hyperparathyroidism, specifically parathyroid adenoma. This was confirmed by a Tc-99m sestamibi scan, and normalization of the calcium level was achieved after parathyroidectomy.

This patient had a single parathyroid adenoma, as evidenced by sestamibi scintigraphy and the presence of a concomitant thyroid nodule by the USG neck. Concomitant thyroid pathology is not uncommon in patients with primary hyperparathyroidism, with findings ranging from 20% to as high as 90% in various studies [5]. Coexistent thyroid disease may complicate the preoperative localization of abnormal parathyroid glands using sestamibi scintigraphy and neck USG. Additionally, sestamibi scintigraphy has poor sensitivity and may miss multigland disease [6], necessitating IOPTH monitoring. The complete clearance of parathyroid adenoma was confirmed by a 50% reduction in IOPTH levels at 10 and 20 minutes post-parathyroid gland removal (Table 2) [7].

PTH has a short half-life of about three to five minutes, making it suitable for intraoperative use [8], and IOPTH monitoring is used to confirm the removal of all hyperfunctioning parathyroid tissue. This allows the surgeon to terminate the surgery confidently once an appropriate decline in the serum iPTH level is observed [9]. Of note, persistent elevation of serum iPTH levels after gland removal necessitates further exploration by the surgeon for other sources of PTH, such as in a multigland disease or ectopically located glands. Given that this patient has chronic renal insufficiency, an IOPTH measurement at 15 minutes post-parathyroid gland removal is recommended because of a slower decline in the iPTH level and to avoid false-negative results [10].

## Conclusions

Patients with malignant tumors and concomitant hypercalcemia should be evaluated for the possibility of primary hyperparathyroidism. This case highlights the importance of appropriate assessment in hypercalcemic patients with malignancy to avoid complications. If hyperparathyroidism is determined to be the cause of hypercalcemia, parathyroidectomy with IOPTH monitoring is indicated.
